# The Assessment of Post-COVID Fatigue and Its Relationship to the Severity and Duration of Acute COVID Illness

**DOI:** 10.3390/jcm12185910

**Published:** 2023-09-12

**Authors:** Alexandria Muench, Elizabeth W. Lampe, Julia T. Boyle, Mark Seewald, Michelle G. Thompson, Michael L. Perlis, Ivan Vargas

**Affiliations:** 1 Behavioral Sleep Medicine Program, Department of Psychiatry, University of Pennsylvania, 3535 Market Street, Philadelphia, PA 19104, USA; mark.seewald@pennmedicine.upenn.edu (M.S.); mperlis@upenn.edu; 2 Chronobiology and Sleep Institute, Department of Medicine, University of Pennsylvania, Philadelphia, PA 19104, USA; 3 Center for Weight Eating and Lifestyle Sciences, Drexel University, Philadelphia, PA 19104, USA; ewl34@drexel.edu; 4 Department of Psychological and Brain Sciences, Drexel University, Philadelphia, PA 19104, USA; 5 New England Geriatric Research Education and Clinical Center, VA Boston Healthcare System, Boston, MA 02130, USA; julia.boyle@va.gov; 6 Department of Psychiatry, Harvard Medical School, Boston, MA 02115, USA; 7 Annenberg School for Communication, University of Pennsylvania, Philadelphia, PA 19104, USA; michelle.thompson@asc.upenn.edu; 8 Department of Psychological Science, University of Arkansas, Fayetteville, AK 72701, USA; ivvargas@uark.edu

**Keywords:** COVID-19, long COVID, fatigue, stress

## Abstract

Emerging data suggests that COVID-19 is associated with fatigue well beyond the acute illness period. The present analysis aimed to: (1) characterize the prevalence and incidence of high fatigue at baseline and follow-up; (2) examine the impact of COVID-19 diagnosis on fatigue level following acute illness; and (3) examine the impact of acute COVID-19 symptom severity and duration on fatigue at follow-up. Subjects (*n* = 1417; 81.0% female; 83.3% White; $\bar{X}$_age_ = 43.6 years) completed the PROMIS-Fatigue during the initial wave of the pandemic at baseline (April–June 2020) and 9-month follow-up (January–March 2021). A generalized linear model (binomial distribution) was used to examine whether COVID-19 positivity, severity, and duration were associated with higher fatigue level at follow-up. Prevalence of high fatigue at baseline was 21.88% and 22.16% at follow-up, with 8.12% new cases at follow-up. Testing positive for COVID-19 was significantly associated with higher fatigue at follow-up. COVID-19 symptom duration and severity were significantly associated with increased fatigue at follow-up. COVID-19 symptom duration and severity during acute illness may precipitate longer-term fatigue, which could have implications for treatment planning and future research. Future studies should further evaluate the relationship between symptom severity, duration, and fatigue.

## 1. Introduction

In March 2020, the World Health Organization (WHO) declared Coronavirus 2019 (COVID-19), a highly contagious disease caused by severe acute respiratory syndrome coronavirus 2 (SARS-CoV-2) [1], a global pandemic. As of May 2023, there were over 765 million confirmed cases and 6.9 million deaths globally [2]. Symptoms of COVID-19 can vary greatly, with infected persons reporting a myriad of symptoms including cough, fever, dyspnea, headaches, sore throat, or no symptoms at all [3,4,5,6]. A growing body of literature suggests that infection with COVID-19 may increase fatigue levels in affected individuals [7] and may extend post-acute illness [8]. The term “fatigue” is problematic because it is commonly used both as a term in everyday vernacular and within clinical settings. In the vernacular, fatigue refers to transient sensations that range from weariness to exhaustion. Clinically, the term refers to pathological levels of persistent physical, emotional, and/or cognitive “enervation” that appears not to be relieved, or improved by, rest or sleep [9,10]. Generally, the prevalence of fatigue in the United States ranges from 7–45% of the population [11]. Given that fatigue often occurs with infectious diseases (such as influenza), it is not surprising that individuals who contract COVID-19 report fatigue during the acute phase of the illness (approximately one to two weeks). Furthermore, long-term fatigue is also highly prevalent in several chronic conditions, like multiple sclerosis and heart failure, with up to 90% of patients reporting increased fatigue over several months or years [12,13]. Long-term fatigue is associated with poor physical and mental health, and cognitive impairment [14]. 

Acute COVID symptoms may lead to more virulent forms of Post-COVID fatigue [15,16]. In a large scale, long term study of COVID, it was found that 87% of 31,000 patients experienced at least one persistent COVID-19 symptom following acute illness (e.g., anxiety and headaches) [17]. According to one systematic review, fatigue may be one of the most persistent sequelae following hospitalization due to COVID-19 [18]. A more recent meta-analysis on post-COVID-19 fatigue found that potential risk factors for fatigue were older aged patients at diagnosis, female sex, more severe infection, a high number of medical comorbidities, and depression/anxiety [19]. Two recent longitudinal studies provide convergent evidence that persistent fatigue is prevalent amongst those who were hospitalized for COVID-19 with over half of patients (52%) reporting fatigue at six months [20,21]. Despite this growing body of literature, it remains unknown as to what extent the duration and severity of other acute COVID-19 symptoms (e.g., fever or chills, cough & difficulty breathing, loss of taste or smell, etc.) may impact long term levels of fatigue post-acute illness in the general population (i.e., those that were not necessarily hospitalized for COVID-19). The possible mechanisms that have been proffered to explain Long-COVID include impaired cortical excitability, brain perfusion, and immune dysregulation. However, more research is needed to further elucidate what may be causing this syndrome [22,23,24,25]. 

Given the high rate of fatigue following acute COVID-19 illness, it seems evident that the COVID-19 pandemic poses a risk for widespread onset of chronic fatigue in populations across the globe. As such, there is an increased need to better understand the nature of fatigue following the COVID-19 illness. While there is a growing body of literature on the prevalence of post-COVID-19 fatigue, more work remains to be conducted to quantify what may be influencing the maintenance of fatigue post-infection, especially in those who contracted COVID before vaccines were widely available. The aims of the present study were to: (1) characterize the prevalence and incidence of high fatigue at baseline (i.e., very early in the COVID-19 pandemic; April–June 2020) and at follow-up (January–March 2021); (2) examine whether those diagnosed with COVID-19 during the study period endorsed higher fatigue at follow-up (after acute illness); and (3) examine the impact of acute COVID-19 symptom severity and duration on fatigue levels following illness. 

## 2. Materials & Methods

### 2.1. Participants and Procedures

The present study was a secondary analysis from an online survey that assessed sleep, mood, and COVID-19-related symptoms from 4145 adults living in the United States during the COVID-19 pandemic [26]. The parent study was designed to evaluate the effects of social distancing on mood and sleep (*N* = 4145; 78.7% female; $\bar{X}$_age_ = 45.8 years). 

The study was a longitudinal design, and recruitment occurred between March–June 2020, with follow-up in January–March 2021. Recruitment efforts used convenience sampling and included postings on multiple social media websites (e.g., Facebook, Reddit), an email list of former research subjects that had previously completed a sleep screener, online newsletters, and ResearchMatch. Inclusion criteria was as follows: at least 18 years old, internet access, and the ability to read and write in English. Eligible individuals were first invited to complete a baseline survey followed by twice daily brief surveys (one in the morning and one in the evening) for two weeks. Subjects were instructed to complete the morning (AM) survey after waking up and the evening (PM) survey before going to bed. The surveys were administered via Qualtrics XM (Provo, UT, USA). Participants were included in the subsequent analyses if they completed at least one of the daily assessments (range = 1–14 days).

The subsample for the present study includes subjects who completed the PROMIS Short Form—Fatigue 7a at baseline and follow-up (*n* = 1417; 81.0% female; 83.3% White; $\bar{X}$_age_ = 43.6 years). As part of the original study, the PROMIS Fatigue scale was administered twice: baseline = March–June 2020, and follow-up = January–March 2021. Subjects who reported testing positive for COVID-19 also provided additional information on COVID-19 symptom type (e.g., cough, fever) severity, and duration. All procedures were approved by the Institutional Review Boards at the University of Arkansas and University of Pennsylvania.

### 2.2. Measures

Demographics: Subjects were asked to provide their age (in years), select their gender identity (from male, female, transgender male, transgender female, gender variant/non-conforming, and other), and provide their racial identity (from American Indian, Native American, Alaska Native; Asian or Asian American; Black, African American, African; Latino or Latina; Middle Eastern or Arab; Native Hawaiian or Other Pacific Islander; White or Caucasian; Multi-racial; Other). 

COVID-19 Questions: At both baseline and follow-up, subjects were asked to report if they had been tested for COVID-19. They were also asked to report if they tested positive for COVID-19 and, if so, provide additional information on COVID-19 symptoms (e.g., fever, body aches, muscle pain, chills, dry cough), severity (e.g., How severe was your cough?; 0, not severe at all-4, extremely severe), and duration (e.g., How long did your COVID-19 symptoms last? [in days]) during the acute illness phase. 

PROMIS Short Form—Fatigue 7a: Subjects were provided with the PROMIS-Short Form-Fatigue 7a (PROMIS F-SF-7a) at both time points. The PROMIS is a seven-item questionnaire that assesses a range of self-reported fatigue symptoms over the past seven days. The scale is divided into six categories (i.e., frequency, duration, & intensity; impact of fatigue on physical, mental, and social activities) and ranges from mild fatigue to an overwhelming sense of exhaustion. Response options are on a 5-point Likert scale, ranging from 1 (Never) to 5 (Always), with one item being reverse scored. Scores were summed and converted to a T-score ($\bar{X}$ = 50 ± 10) with scores ranging from a 55 (mild fatigue) to 80 (severe fatigue). For the purposes of describing the sample in aim 1 and examining predictors of transition from low-to-high-fatigue groups in aim 3, high fatigue was defined as a PROMIS T-score of ≥60. The threshold to evaluate within-group change or make between-group comparisons is between two and six T-score points [27]. PROMIS total score was treated as a continuous variable for aim 2. The PROMIS F-SF has been evaluated in many different populations [28,29]. The measure demonstrates good internal consistency reliability (Cronbach’s α = 0.84) [30]. 

### 2.3. Statistical Analyses

Statistical analyses were completed in R (version 4.1.3) [31]. Alpha ≤ 0.05 was used to draw inferences of statistical significance across all models tested. Data were explored for extreme values, and no statistical or logical outliers were identified. All analyses included the 1417 subjects who completed the PROMIS screener at both baseline and follow-up.

Aim 1. Fatigue prevalence and incidence rates. Fatigue group was independently coded as 0 [low fatigue] and 1 [high fatigue] at both baseline and follow-up. The proportion of subjects who met the criteria for high fatigue on the PROMIS at baseline and follow-up was calculated. Next, a series of chi-square tests were used to estimate whether COVID-19 testing, and positivity was related to baseline fatigue. 

Aims 2 and 3. Impact of COVID-19 diagnosis, and symptom severity/fatigue on fatigue at follow-up. A series of linear regressions were used to examine whether COVID-19 positivity, symptom duration and severity were associated with increased follow-up fatigue while controlling for baseline fatigue. A generalized linear model with a binomial distribution was used to examine whether COVID-19 positivity, severity, and duration were associated with transition from the low-fatigue group at baseline to the high-fatigue group at follow-up. COVID-19 testing and positivity rates were coded as binary variables (no [0]/yes [1]). COVID-19 symptom duration and severity were treated as continuous variables and entered as independent variables in separate models. Analyses also adjusted for covariates that were significantly associated with baseline fatigue (e.g., age, gender, educational history, employment status and history of chronic illnesses; see Table 1).

## 3. Results

### 3.1. Participant Demographics and Baseline Differences

Table 1 summarizes means, standard deviations and percentiles for all demographic variables and whether there were any group differences at baseline. According to these data, the high-fatigue group (as determined by baseline PROMIS scores) was slightly younger, less likely to identify as a cis-gender female or cis-gender male, had a lower level of education, and were less likely to be employed, and less likely to report having a history of at least one chronic illness (e.g., cardiovascular disease, diabetes).

### 3.2. Prevalence and Incidence of High Fatigue at Baseline and Follow-Up

**Figure 1:**
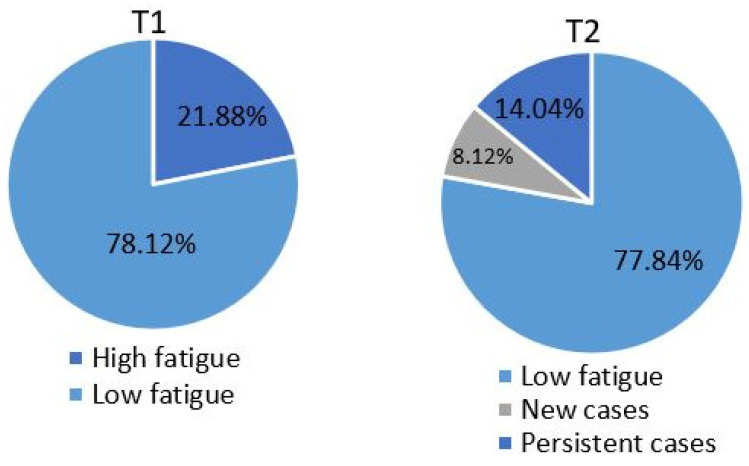
The proportion of individuals categorized as low fatigue, high fatigue and new/persistent cases of high fatigue at baseline (T1) and follow-up (T2).

According to the data collected at baseline, the sample mean score on the PROMIS was 52.85 (SD = 9.37). The prevalence of high levels of fatigue at baseline (PROMIS T-score ≥ 60) was 21.88% (*n* = 310). The sample mean at follow-up was 52.81 (SD = 9.37). The overall prevalence of high fatigue at follow-up was 22.16% (*n* = 314). Of those that endorsed clinical levels of fatigue at baseline, 7.83% (*n* = 111) no longer met criteria for clinical fatigue at follow-up. In contrast, the incidence of clinical fatigue (i.e., percent of new cases at follow-up) was 8.12% (*n* = 115; Figure 1).

### 3.3. COVID-19 Testing and Positivity Rates

Among those who completed the PROMIS at baseline (*n* = 1417), 12.14% of the sample reported getting tested for COVID-19 (*n* = 172) during the study period. Among those that reported getting tested, 126 participants (8.89%) reported testing positive for COVID-19. While the rate of testing for COVID-19 during the study period did not vary by baseline fatigue group, high fatigue = 43 (3.03%) vs. low fatigue = 129 (9.10%), χ^2^ < 0.99, *p* = 0.32, those with higher baseline fatigue were more likely to report testing positive for COVID-19 during the study period (*t*(1416) = 208.25, *p* < 0.001; M_positive_ = 55.83 [SD = 7.86], M_negative_ = 52.85 [SD = 9.39]).

### 3.4. Effects of COVID-19 on Fatigue at Follow-Up

Testing positive for COVID-19 during the study period was significantly associated with increased fatigue at follow-up (B = 1.88, S.E. = 0.60, *p* = 0.002, f^2^ = 0.06) with a small effect size when controlling for baseline fatigue. Only those participants who tested positive for COVID-19 during the study period (*n* = 149) were included in analyses examining the effect of acute COVID-19 symptom severity and duration on follow-up fatigue. Both acute COVID-19 symptom duration (B = 0.05, S.E. = 0.03, *p* = 0.037, f^2^ = 0.13) and symptom severity (B = 1.30, S.E. = 0.52, *p* = 0.015, f^2^ = 0.15) were significantly associated with increased fatigue at follow-up when controlling for baseline fatigue with small-to-medium effect sizes (Table 2). COVID-19 positivity, severity, and duration were not significantly associated with transition from the low-fatigue group at baseline to the high-fatigue group at follow-up (Table 3).

## 4. Discussion

In this national study of adults living in the United States who were surveyed during the COVID-19 pandemic, the findings indicated that: (1) there was a significant positive association between acute COVID-19 positivity, severity, and duration and fatigue levels at follow-up; (2) those with higher fatigue at baseline were more likely to contract COVID-19 during the study; and (3) at baseline, the high-fatigue group was younger, of a lower SES, and less educated.

The association between COVID-19 positivity, severity, and duration may be the most interesting and promising finding from this study. It seems to be the case that, while simply testing positive for COVID-19 was associated with increased fatigue, greater self-reported symptom severity and duration during the acute phase of illness further increased one’s chances of reporting high fatigue at follow-up. Corroborating research conducted by Hanson and colleagues (2022) [32] evaluated the occurrence, severity, and recovery pattern of 1906 community infections and 10,526 hospitalized patients from the ten collaborating cohorts, where more severe, hospitalized patients were more likely to report persistence of COVID-19 symptoms.

With respect to higher fatigue at baseline predicting COVID-19 infection, it was well-documented in the literature that fatigue and the immune system are intricately linked, thus it may be that those who reported higher fatigue at baseline were more susceptible to infection. For example, one study used PET/MR imaging to evaluate two independent biomarkers thought to be related to fatigue in 57 healthy individuals. The authors found PET signal increases that were associated with physical and mental fatigue and elevated inflammatory makers that were correlated with the neuroimaging markers [33]. Notably, those who had higher fatigue at baseline were more likely to be younger, have a lower SES, and fewer years of education. These groups make up a large portion of those who were required to keep working outside the home during lockdowns and likely would have had higher chances of exposure to COVID-19, thus making them more likely to report testing positive for COVID-19 at follow-up. Moreover, similar findings were found by Frontera et al. (2021) [34]. One thousand subjects who were diagnosed with COVID-19 completed self-report measures of fatigue and psychiatric symptoms, where symptoms were found to be of greater duration. The patients included in this study were often younger, female, Hispanic, had a history of anxiety and/or depression, and were either unemployed and/or reporting financial insecurity.

The findings of the current study, combined with those in the literature, may be the result of the relationship between fatigue and chronic inflammation, which makes individuals more vulnerable to a greater response to infection. These results may have implications for clinical practice and future COVID-19 research. Clinicians and healthcare professionals may wish to periodically assess fatigue in patients who test positive for COVID-19. Brief, primary-care-friendly assessments, such as the PROMIS measure used in the present study, may be useful in assessing symptoms over time. Regarding COVID-19 research, investigators may wish to include a brief fatigue measure in their methodology. While this may not be a primary outcome of all COVID-19 research, it would be important to consider for controlling fatigue, especially over time.

### Strengths and Limitations

While the findings from the current study are preliminary, they are an important first step in delineating not just the differences in fatigue between those who are and are not diagnosed with COVID-19, but also the lasting negative impact that acute COVID-19 illness may have on mental health and overall quality of life by increasing fatigue levels. Moreover, the results may be important when considering treatment planning for patients who have been diagnosed with COVID-19. With respect to preventing long-term fatigue, it may be important to identify patients with longer/more severe symptoms as early as possible, as this would allow for targeted intervention to reduce fatigue following acute illness.

While this study has many strengths, there are a few limitations. First, while also a potential strength, this study was conducted during the initial wave of the pandemic. While this allowed us to isolate the effect of COVID-19 on fatigue prior to the widespread availability of vaccinations (which were first implemented in January of 2021), since then, there have been more than two different variants with different symptoms and severity. Thus, one could conclude that fatigue may present differently, due to differences in the presentation of the variants. Further, we did not collect information on variants of COVID-19, as this information was not widely available to patients at the time. It is possible that there was a significant difference between baseline and follow-up in the proportion of variant type among COVID-19-positive participants. Second, despite finding that COVID-19 positivity, severity, and duration were all associated with increased fatigue at follow-up, the uncertain nature of the early pandemic may have led to elevated fatigue scores in the non-COVID group. Third, the advent of COVID-19 vaccines may decrease the risk of elevated fatigue post-acute COVID-19 infection, however, that has yet to be determined. Third, subjects may have been still experiencing symptoms when they filled out the follow-up survey, which may have impacted the study findings. Fourth, this study relied on self-report assessment of COVID-19 positivity, as well as symptom severity and duration. There may have been larger differences if an objective measure of COVID-19 positivity, such as a PCR test, was used. Similarly, the current study relied on “subjective” self-report of illness severity and duration. More objective indicators of disease severity (e.g., hospital admission, supplementary oxygen administration, etc.) would provide even more information, which could be used to inform risk-models of post-COVID fatigue. Fifth, the sample included was likely not representative of the general population (i.e., more than 80% white women), which limits generalizability. Sixth, we did not assess cognitive impairment associated with fatigue in this sample. Future work should explore the effects of COVID-19 and post-COVID fatigue on cognitive impairment. Finally, there was a subset of subjects who completed the study before the fatigue measure was added, which may have affected the results.

## 5. Conclusions

There is little research on the long-term impacts of acute COVID-19 illness, especially in those individuals who developed COVID-19 early in the pandemic and prior to the availability of vaccines. This research sheds light on the possible chronic effects of acute COVID-19 illness and the need to follow-up post-acute infection. Future research may wish to examine differences in post-COVID fatigue amongst specific variants of the SARS-CoV-2 virus, and in those who are vaccinated, as an outcome of pandemic-related impacts on mental and physical health.

## Figures and Tables

**Table 1:** Participant Demographic Information by Fatigue Status. *p*-values were determined using *t*-tests for continuous variables and chi-square analyses for categorical variables. * p ≤ 0.05.

	Total Sample (*n* = 1417)	High Fatigue (*n* = 310)	Low Fatigue (*n* = 1107)	*p*-Value
Age, mean (SD)	43.63 (16.58)	40.62 (15.24)	44.48 (16.85)	<0.001 *
Gender, *n* (%)				<0.001 *
Female	1148 (81.02)	258 (83.23)	890 (80.40)	
Male	237 (16.73)	34 (10.97)	203 (18.34)	
Transgender Female	2 (0.14)	0 (0)	2 (0.18)	
Transgender Male	4 (0.28)	2 (0.65)	2 (0.18)	
Gender Variant/Non-Conforming	18 (1.27)	13 (4.19)	5 (0.45)	
Other	8 (0.62)	3 (0.97)	5 (0.45)	
Race/Ethnicity, *n* (%)				0.29
American Indian, Native American, or Alaska Native	2 (0.14)	1 (0.32)	1 (0.0001)	
Asian or Asian American	66 (4.65)	9 (2.90)	57 (5.15)	
Black, African American, or African	57 (4.02)	17 (5.48)	40 (3.61)	
Latino or Latina	46 (3.25)	12 (3.87)	34 (3.07)	
Middle Eastern or Arab	7 (0.49)	2 (0.65)	5 (0.45)	
Native Hawaiian or Other Pacific Islander	3 (0.21)	1 (0.32)	0 (0)	
White or Caucasian	1180 (83.27)	251 (80.97)	929 (83.92)	
Multi-racial	43 (3.03)	14 (4.52)	29 (2.62)	
Other	13 (0.92)	3 (0.97)	10 (0.90)	
Marital Status, *n* (%)				0.15
Married	589 (41.57)	110 (35.48)	479 (43.27)	
Widowed	43 (3.03)	8 (2.58)	35 (3.16)	
Divorced	171 (12.07)	42 (13.55)	129 (11.65)	
Separated	13 (0.92)	3 (0.97)	10 (0.90)	
Never Married	600 (42.34)	146 (47.10)	454 (41.01)	
Educational History, *n* (%)				<0.001 *
less than high school	4 (0.28)	0 (0)	4 (0.36)	
high school graduate	46 (3.25)	11 (3.55)	35 (3.16)	
some college	152 (10.73)	49 (15.81)	103 (9.30)	
2-year degree	97 (6.85)	30 (9.68)	67 (6.05)	
4-year degree	529 (37.33)	120 (31.71)	409 (36.95)	
professional degree	476 (33.59)	75 (24.19)	401 (36.22)	
doctorate	113 (7.97)	25 (8.06)	88 (7.95)	
Employment, *n* (%)				0.02 *
Unemployed	467 (32.96)	123 (39.68)	344 (31.07)	
employed 1–20 h	139 (9.81)	24 (7.74)	115 (37.10)	
employed 20–30 h	104 (7.34)	17 (5.48)	87 (7.86)	
employed full time (40+ hours)	700 (49.40)	145 (46.77)	555 (50.14)	
Chronic Illness, *n* (%)				<0.001 *
No	682 (48.13)	115 (37.10)	567 (51.22)	
Yes	735 (51.87)	195 (62.90)	540 (48.78)	

**Table 2:** Results of linear models examining the effect of acute COVID-19 positivity, severity, and duration on fatigue at follow-up. * p ≤ 0.05.

Predictor	B	S.E.	t	*p*-Value	f^2^	95% C.I. for f^2^
Positive COVID-19 test	1.88	0.60	3.13	0.002 *	0.06	0.02–0.09
COVID-19 Symptom Severity	1.30	0.52	2.48	0.015 *	0.15	0.03–0.26
COVID-19 Symptom Duration	0.05	0.03	2.11	0.037 *	0.13	0.01–0.24

**Table 3:** Results of generalized linear models examining the effect of acute COVID-19 positivity, severity, and duration on categorization in the high-fatigue group at follow-up.

Predictor	B	S.E.	z	*p*-Value	OR	95% C.I. for OR
Positive COVID-19 test	0.162	0.324	0.499	0.618	0.05	−0.15–0.22
COVID-19 Symptom Severity	0.558	0.326	1.709	0.088	0.56	−0.07–1.25
COVID-19 Symptom Duration	0.023	0.014	1.675	0.094	0.48	−0.12–1.04

## Data Availability

The data underlying this article will be shared on reasonable request to the corresponding author.
